# Gnetin C Intercepts MTA1-Associated Neoplastic Progression in Prostate Cancer

**DOI:** 10.3390/cancers14246038

**Published:** 2022-12-08

**Authors:** Prashanth Parupathi, Gisella Campanelli, Rabab Al Deabel, Anand Puaar, Lakshmi Sirisha Devarakonda, Avinash Kumar, Anait S. Levenson

**Affiliations:** 1 Division of Pharmaceutical Sciences, Arnold & Marie Schwartz College of Pharmacy and Health Sciences, Long Island University, Brooklyn, NY 11201, USA; 2 Department of Biomedical Sciences, School of Health Professions and Nursing, Long Island University, Brookville, NY 11548, USA; 3 Department of Biomedical Sciences, College of Veterinary Medicine, Long Island University, Brookville, NY 11548, USA

**Keywords:** gnetin C, diet supplementation, transgenic mice, targeted interception, MTA1, prostate cancer

## Abstract

**Simple Summary:**

The incidence of prostate cancer is increasing because of the aging population. Evidence suggests that diets rich in bioactive polyphenols can reduce the incidence of prostate cancer. The aim of this work was to investigate the potential of gnetin C, a compound found in the melinjo plant and commonly used in Indonesian foods, to block prostate cancer progression. To this end, we evaluated the anticancer efficacy of gnetin C-supplemented diets in a unique and adequate high-risk premalignant prostate cancer transgenic mouse model. Our results indicate that a gnetin C-supplemented diet reduces the progression of prostate cancer by reducing the proliferation of cells, inflammation, and the formation of blood vessels. The finding that a gnetin C-supplemented diet effectively blocks tumor progression in a preclinical mouse model may be exploited to initiate chemoprevention trials for novel nutritional interception for untreated patients under active surveillance.

**Abstract:**

Nutritional chemoprevention is particularly suitable for prostate cancer. Gnetin C, a resveratrol dimer found abundantly in the melinjo plant (*Gnetum gnemon*), may possess more potent biological properties compared to other stilbenes. We examined the effects of gnetin C in a high-risk premalignant transgenic mouse model overexpressing tumor-promoting metastasis-associated protein 1 (MTA1) on the background of *Pten* heterozygosity (*R26^MTA1^*; *Pten^+/f^*; *Pb-Cre^+^*). Mice were fed diets supplemented with the following compounds: pterostilbene (70 mg/kg diet); gnetin C, high dose (70 mg/kg diet); and gnetin C, low dose (35 mg/kg diet). Prostate tissues were isolated after 17 weeks and examined for histopathology and molecular markers. Serum was analyzed for cytokine expression. Gnetin C-supplemented diets substantially delayed the progression of preneoplastic lesions compared to other groups. Prostate tissues from gnetin C-fed mice showed favorable histopathology, with decreased severity and number of prostatic intraepithelial neoplasia (PIN) foci, reduced proliferation, and angiogenesis. A decreased level of MTA1, concurrent with the trend of increasing phosphatase and tensin homolog expression and reduced interleukin 2 (IL-2) levels in sera, were also detected in gnetin C-fed mice. Importantly, gnetin C did not exert any visible toxicity in mice. Our findings demonstrate that a gnetin C-supplemented diet effectively blocks MTA1-promoted tumor progression activity in high-risk premalignant prostate cancer, which indicates its potential as a novel form of nutritional interception for prostate cancer chemoprevention.

## 1. Introduction 

Prostate cancer remains the second highest type of cancer-related mortality in men [1]. According to the American Cancer Society, prostate cancer accounts for about 14% of newly diagnosed cancers, and about 5.7% of all cancer deaths in the United States. Although prostate cancer incidence remained stable over the past few years, there was an annual 4–6% increase in advanced prostate cancer, which accounted for the rise in cases of metastatic disease [2]. Recent updates from the National Comprehensive Cancer Network guidelines for prostate cancer highlight a new risk categorization system for patients with prostate cancer, which demands different approaches for the management of the disease [3]. For the three categories defined as “very low”, “low”, and “intermediate” risk groups, there is no treatment strategy in current practice to prevent prostate cancer progression. It has been accepted that diverse clinical lesions in these groups are represented by “large gland” morphology, including high-grade prostatic intraepithelial neoplasia (PIN) and PIN-like carcinoma, which are main precursors to invasive carcinoma [4]. We believe that nutritional interception may represent the most adequate intervention to protect not only the general population, but also the moderate- or high-risk subpopulation of patients under active surveillance.

Epidemiological studies have continually supported the argument that naturally occurring dietary polyphenols with anti-inflammatory, antioxidant, and anticancer properties may be considered for prostate cancer chemoprevention [5,6,7,8]. It is well-established that natural polyphenols of different classes have pleiotropic effects through various signaling pathways, including epigenetic mechanisms, in inhibiting the progression of prostate cancer [9,10,11]. Specifically, the potential application of stilbene polyphenols acting through multiple mechanisms in prostate cancer chemoprevention and treatment was demonstrated and summarized by several groups [12,13,14,15,16,17].

**Figure 1:**
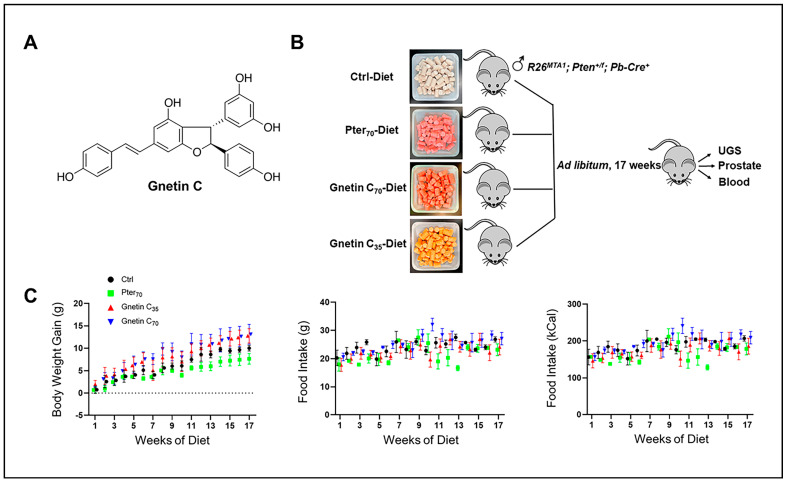
(**A**) Chemical structure of gnetin C, a resveratrol dimer, isolated from melinjo plant (*Gnetum gnemon*). (**B**) Schema showing the experimental design used for prostate cancer chemoprevention by diet supplementation in a precancerous *R26^MTA1^*; *Pten^+/f^*; *Cre^+^* murine model. In total, 24 mice were fed with the control diet or diets supplemented with Pter_70_ (70 mg/kg diet), Gnetin C_70_ (70 mg/kg diet), or Gnetin C_35_ (35 mg/kg diet). At sacrifice, urogenital system and prostate tissues were isolated for histological and molecular analysis. Blood was also collected. (**C**) Effects of different diets on average body weight gain and food intake in mice. Values are mean ± SD, *n* = 6 per group.

It is imperative to establish a relevant and adequate preclinical model system that represents heterogeneous lesions observed in the clinic, in which to determine the efficacy of natural compounds [18]. In our previous studies, we have shown that stilbenes, such as resveratrol and pterostilbene, can act through metastasis-associated protein 1 (MTA1)-mediated mechanisms to prevent the progression of premalignant prostate cancer to adenocarcinoma [19,20,21,22,23,24,25,26,27]. MTA1 is a chromatin modifier and transcriptional regulator, and plays a cancer-promoting role in all stages of prostate cancer [19,27,28,29]. Aberrant alterations in the molecular levels of MTA1 trigger several downstream targets with significant roles in inflammation, cell survival, and invasion, ultimately causing metastasis [22,26,30,31]. Among the downstream pathways affected by the changes in MTA1 levels is the phosphatase and tensin homolog/v-akt murine thymoma viral oncogene (protein kinase B) (PTEN/Akt) pathway, the deregulation of which plays an essential role in prostate cancer [22,32,33]. We have previously shown an inverse association of MTA1 with PTEN, and a direct correlation of MTA1 with p-Akt [22,34]. In fact, we have demonstrated that MTA1 inhibition by resveratrol and pterostilbene promotes the acetylation and activity of PTEN, which in turn inhibits the activity of Akt [22,34]. Given that MTA1 can be targeted by stilbene polyphenols, these compounds are of great interest. We have recently reported that a pterostilbene-supplemented diet exerted beneficial effects by diminishing inflammatory pathways and accelerated PIN progression in *R26^MTA1^*; *Pten^+/f^* mice [27]. Since we have also previously demonstrated the more potent MTA1-targeted inhibitory activity of gnetin C, a dimer resveratrol (Figure 1A), compared to resveratrol and pterostilbene, both in vitro and in prostate cancer xenografts [31,35], we sought to determine the efficacy of gnetin C in an adequate preclinical model of prostate cancer. 

We hypothesized that gnetin C may possess more potent biological effects compared to pterostilbene in a transgenic model of prostate cancer. Therefore, the current study was undertaken to examine the promising MTA1/PTEN/Akt-mediated chemopreventive and interceptive properties of gnetin C, a dimeric stilbenoid, using a clinically relevant model of murine prostate cancer representing high-risk premalignant neoplasia.

## 2. Results

### 2.1. Effects of Gnetin C-Supplemented Diet on Prostate Cancer Progression 

**Figure 2:**
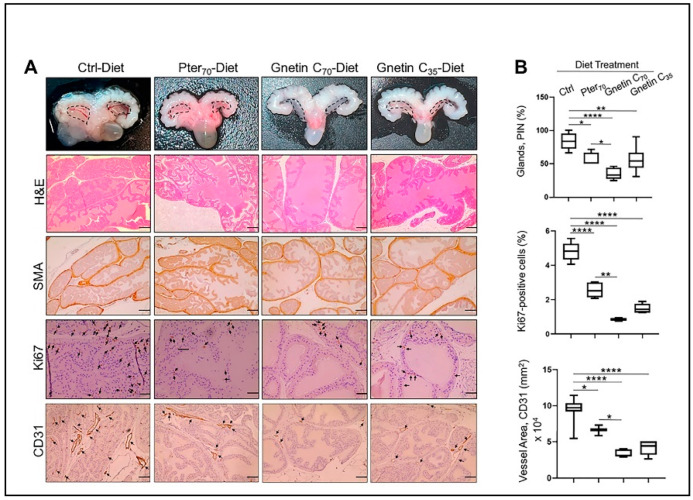
(**A**) *Top*, Representative images of UGS of mice from different groups: The anterior prostates (marked) in mice fed regular diet were larger compared to the treatment groups, but there were no differences in size between treatment groups. Representative images of H&E (scale bar, 100 μm), SMA (scale bar, 50 μm), and Ki67- and CD31-stained sections (scale bar, 20 μm) of the prostate tissues from mice in different groups. (**B**) *Top*, Quantitation of prostate glands involved in PIN formation: The glands were quantified in five randomly selected areas per sample (*n* = 3 per group), and the average count is expressed as a percent. *Middle*, Quantitative analysis of Ki67 immunostaining, expressed as a percent, showing the drastic effect of gnetin C supplementation on cell proliferation. *Bottom*, Quantitative analysis of CD31 immunostaining, expressed as area, showing the strong effect of gnetin C supplementation on angiogenesis. Values are mean ± SEM analyzed from five separate areas per sample. * *p* < 0.05; ** *p* < 0.01; **** *p* < 0.0001 (one-way ANOVA).

For the current study we accumulated twenty-four *R26^MTA1^*; *Pten^+/f^* mice and randomized them into four groups (*n* = 6 per group) on the respective diets ad libitum for 17 weeks (Figure 1B), after which prostate tissues and blood samples were collected for analysis. Diet supplementation with either pterostilbene or gnetin C did not have any significant effect on either the body weight gain or food intake in these mice (Figure 1C). The effect of such treatment on the gross anatomy of the urogenital system (UGS) over this period is shown in Figure 2A, *upper panel*. Gnetin C-supplemented diets decreased the appearance of the prostate compared to the pterostilbene diet (Pter_70_-Diet)- and control diet (Ctrl-Diet)-fed mice. Consistent with our previous study [27], *R26^MTA1^*; *Pten^+/f^* mice in all the groups developed high-grade PIN characterized by disorganized glandular structures with pseudostratified epithelium and hyperproliferation, but with an intact basal layer of smooth muscle actin (SMA)-positive cells (Figure 2A). However, there were significant differences in pathological features, and some morphological differences, among the groups. As shown in Figure 2A, presenting hematoxylin and eosin (H&E) images, mice fed diets supplemented with compounds demonstrated favorable histopathology, with restored normal ductal structures and fewer glands involved in PIN compared to mice on the Ctrl-Diet (Figure 2B, *top*). Moreover, mice on the gnetin C high-concentration diet (Gnetin C_70_-Diet) exhibited a significantly reduced number of glands involved in PIN compared to the Pter_70_-Diet, suggesting a more potent efficacy of gnetin C in restoring the histopathology of the prostate when used at the same concentration in the diet. The gnetin C low-concentration diet (Gnetin C_35_-Diet) worked at the same level as the Pter_70_-Diet, demonstrating, once more, the greater biological potency of gnetin C compared to pterostilbene. The changes in histopathology were accompanied by a corresponding reduction in proliferation and angiogenesis in treatment groups. Analysis of prostate tissues for the cellular protein marker of proliferation (Ki67) and cluster of differentiation (CD31) markers revealed a significant reduction in corresponding positively stained cells in all treatment groups compared to control mice (Figure 2A, *lower panels*)**.** Importantly, once again, the differences between reduced proliferation and angiogenesis in mice of the Gnetin C_70_-Diet and Pter_70_-Diet groups were statistically significant (Figure 2B, *lower panels*). Notably, gnetin C at half concentration (Gnetin C_35_-Diet) showed either the same or more potency compared to Pter_70_-Diet, indicating that gnetin C is more efficacious than pterostilbene. However, there were no significant differences in the anticancer histopathological activities between the lower and higher concentrations of gnetin C diets. Taken together, our data indicate a more potent anticancer activity of gnetin C compared to pterostilbene when provided as dietary supplementation in mice with premalignant neoplasia. 

### 2.2. Gnetin C Effectively Inhibits the MTA1-Associated PTEN/Akt Axis in a Transgenic Mouse Model of Early-Stage Prostate Cancer

**Figure 3:**
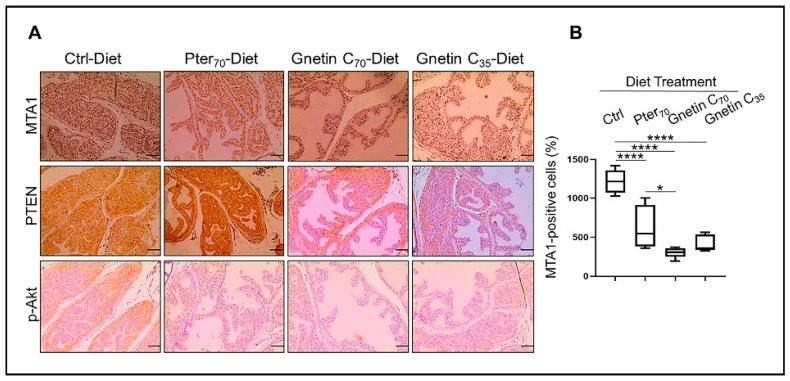
(**A**) Representative images of MTA1 (*top*), PTEN (*middle*), and p-Akt (*bottom*)-stained sections of the prostate tissues from mice in different groups (*n* = 3 per group). Images are 20× (scale bar, 50 µm). (**B**) Quantitative analysis of MTA1 immunostaining. Values are mean ± SEM of cells counted in five separate areas per sample, and the average count is expressed as a percent. * *p* < 0.05; **** *p* < 0.0001 (one-way ANOVA).

Our earlier studies using gnetin C in prostate cancer cell lines and xenografts had shown potent inhibition of the MTA1 and MTA1-associated downstream signaling targets, including proto-oncogene 2 (ETS2), Cyclin D1, and Notch 2 [31,35]. To further strengthen our previous in vivo findings on the effects of stilbenes on the MTA1/PTEN/p-Akt axis [22,34], we evaluated the expression of MTA1 and PTEN/pAkt in prostate tissues from *R26^MTA1^*; *Pten^+/f^* mice fed gnetin C diets. We found that MTA1 protein levels were significantly reduced in prostate tissues by diets supplemented with both pterostilbene and gnetin C (Figure 3A *top***,** B*)*. Specifically, both pterostilbene and gnetin C diets inhibited MTA1 expression in prostate tissues compared to control prostates with high significance (*p* < 0.0001) (Figure 3B). Moreover, despite the heterogeneity of MTA1-stained tissues in the Pter_70_-Diet group, the differences between Pter_70_-Diet and Gnetin C_70_-Diet groups were statistically significant. Furthermore, cytoplasmic PTEN and p-Akt staining of tissues showed a concomitant PTEN increase and p-Akt reduction in mice fed with supplemented diets compared to mice fed the control diet. Subtle differences in PTEN and p-Akt levels between the treatment groups are also evident in selected immunochemistry (IHC) images (Figure 3A, *middle and bottom panels*). 

**Figure 4:**
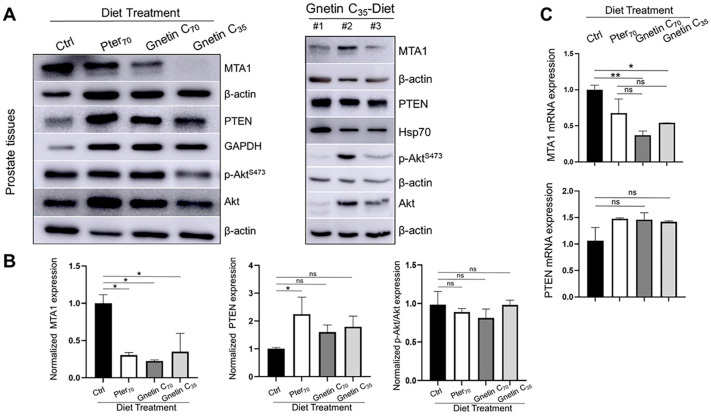
(**A**) Representative immunoblot images of MTA1, PTEN, and p-Akt/Akt levels in the prostate tissues from mice in different treatment groups (*n* = 3 per group). β-actin, GAPDH, or Hsp70 were used as loading controls (*left*). Immunoblot images of MTA1, PTEN, and pAkt/Akt levels in tissues from three mice (#1–3) of the Gnetin C_35_-Diet group (*right*). (**B**) Quantitation of the relative expression of these markers in prostate tissues from mice in different treatment groups (*left*). Values are mean ± SEM of data from three or more independent experiments. * *p* < 0.05; ** *p* < 0.01; (one-way ANOVA). (**C**) Quantitation of relative MTA1 and PTEN mRNA levels in prostate tissues from mice in different treatment groups. β-actin amplification was used as a normalization control. Changes in mRNA expression were calculated by the 2^−ΔΔCt^ method. Values are mean ± SEM of data from three independent experiments. * *p* < 0.05; ** *p* < 0.01; (one-way ANOVA). ns, not significant.

Next, we evaluated the response to treatments by measuring levels of MTA1, PTEN, and p-Akt/Akt in prostate tissue lysates. The expression MTA1 protein levels in prostate tissues from mice fed diets supplemented with stilbenes was significantly reduced compared to control mice (Figure 4A,B). We were also able to detect differences among treatment groups, indicating the trend towards gnetin C’s greater potency. In contrast to the inhibitory effects on MTA1, diets supplemented with pterostilbene and gnetin C restored PTEN levels in prostate tissues (Figure 4A,B). In general, prostate tissues from mice in the same treatment group demonstrated an expected trend compared to control mice, however, with noticeable heterogeneity, particularly with respect to p-Akt/Akt in mice in the Gnetin_35_-Diet group (Figure 4A, *right*) (Supplementary Figure S1). At the messenger RNA (mRNA) level, MTA1 and PTEN were respectively inhibited and upregulated by stilbene-supplemented diets compared to control mice (Figure 4C). The differences among treatments groups were not significant. 

**Figure 5:**
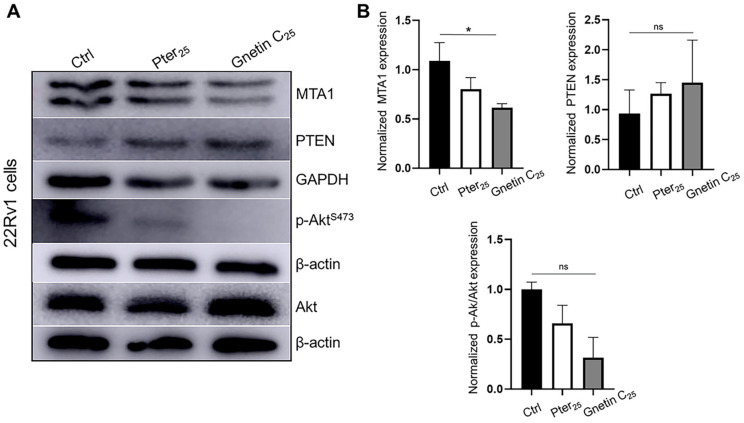
(**A**) Representative immunoblot images of MTA1, PTEN, and p-Akt/Akt levels in 22Rv1 prostate cancer cells treated with pterostilbene and gnetin C at 25 μM concentration. β-actin was used as a loading control. (**B**) Quantitation of the relative expression of these markers in prostate cancer cells. Values are mean ± SEM of data from three independent experiments. * *p* < 0.05; (one-way ANOVA). ns, not significant.

We have chosen the human prostate cancer cell line 22Rv1 that expresses wild-type PTEN for our in vitro experiments. Treatment of these cells for 24 h with the same equipotent concentrations resulted in a statistically significant reduction in MTA1 by gnetin C (*p* < 0.05) and an insignificant increase in PTEN by both compounds compared to control untreated cells, revealing, once again, the more potent activity of gnetin C compared to pterostilbene (Figure 5A,B). Inhibition of p-Akt/Akt under the treatments followed the same trend as MTA1. 

Collectively, these data demonstrate a more potent MTA1/PTEN/Akt response to gnetin C than to pterostilbene treatment, both in a murine prostate model of early-stage prostate cancer and in a prostate cancer cell line. This emphasizes that MTA1-targeted interception by diet supplemented with gnetin C may have greater potential benefits compared to pterostilbene supplementation, which we have recently reported for prostate cancer chemoprevention [27]. 

### 2.3. Effects of Gnetin C-Supplemented Diet on Pro-Inflammatory Interleukin 2 (IL-2) and Interleukin 6 (IL-6) Cytokine Levels in Murine Serum 

**Figure 6:**
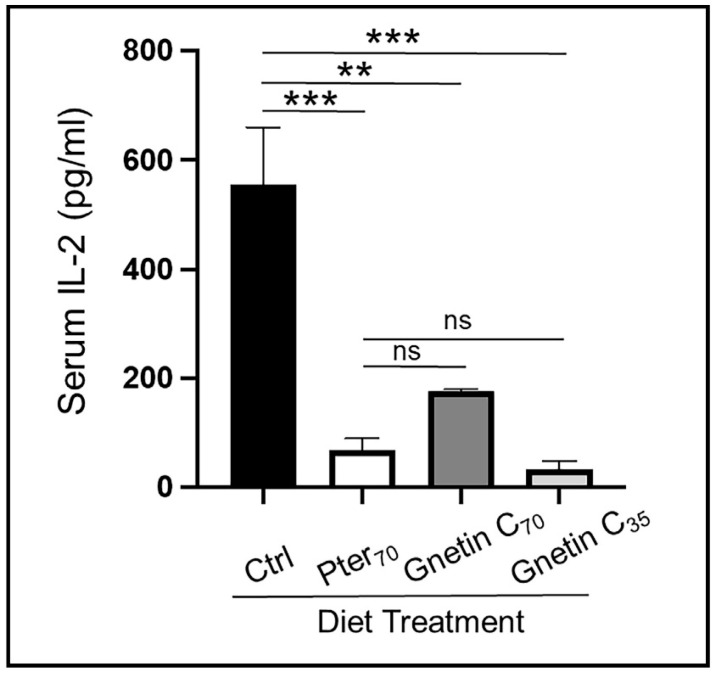
Effects of diets supplemented with gnetin C on circulating IL-2 cytokine levels **measured by ELISA in murine sera** (*n* = 3–4 per group) Data represent the mean ± SEM of three independent experiments performed in duplicate. ** *p* < 0.01; *** *p* < 0.001; (one-way ANOVA). ns, not significant.

To evaluate the systemic efficacy of gnetin C dietary intervention, we determined pro-inflammatory IL-2 and IL-6 levels in mouse sera by enzyme-linked immunosorbent assay (ELISA). Our results show significantly reduced levels of circulating IL-2 in sera of mice fed supplemented diets compared to control mice (68.34% reduction for Gnetin C_70_-Diet and 93.86% reduction for Gnetin C_35_-Diet) (Figure 6A). Interestingly, gnetin C supplementation at the lower 35 mg/kg diet concentration secured a greater attenuation of IL-2 levels compared to both Pter_70_ and Gnetin C_70_ diets. Moreover, the Gnetin C_35_ diet also inhibited IL-6 levels, while Gnetin C_70_-Diet mice showed a paradoxical increase in the levels of pro-inflammatory IL-6 (Supplementary Figure S2). In agreement with our previous data, which revealed that high-concentration pterostilbene supplementation (100 mg/kg diet) reduced serum IL-6 significantly (*p* < 0.01) [27], we now observe a downregulation of IL-6 in mice treated with Pter_70_-Diet, albeit without significance. Nevertheless, these data suggest that systemic inflammation was decreased in mice fed the low-concentration gnetin C-supplemented diet (Gnetin C_35_-Diet), which should ensure the beneficial anti-inflammatory effects of gnetin C, and validate the utilization of sera as a liquid biopsy strategy for the evaluation of gnetin C responsive, prognostic, and predictive noninvasive biomarkers. 

## 3. Discussion

Naturally occurring resveratrol oligomers, including gnetin C, have been proposed as potential cancer chemopreventive compounds [36]. Antitumor activities of gnetin C, a resveratrol dimer, have been reported in acute myeloid leukemia [37], colon cancer [17], and neuroblastoma [38]. Accumulated data indicate that gnetin C exhibits potent antitumor activity in prostate cancer. Using a panel of human prostate cancer cell lines (PC3, LNCaP, and DU145) and mouse prostate cancer cells derived from the adenocarcinoma of PTEN null mice (PTEN-CaP8), Narayanan et al. [17] first reported the significant proliferation inhibitory effects of gnetin C in cancer cells without affecting normal prostate epithelial RWPE-1 cells. Moreover, gnetin C was significantly more potent in inhibiting cell proliferation and apoptosis in prostate cancer cells compared to resveratrol [17]. Our own published in vitro studies with DU145 and PC3M cells showed more potent inhibition of cell proliferation with lower IC_50_ values for gnetin C compared to pterostilbene and resveratrol [35]. In fact, we have repeatedly shown gnetin C-concentration-dependent cell survival inhibition in a panel of prostate cancer cells (Supplemental Figure S3). Moreover, we have demonstrated that gnetin C was more potent in causing apoptosis and inhibiting metastatic potential of prostate cancer cells than resveratrol and pterostilbene [35]. In addition, we also demonstrated that gnetin C is a lead compound among stilbenes for effectively blocking tumor progression in immunodeficient mice implanted with PC3M [35]. Importantly, in a recent paper using genetically modified DU145 and PC3M prostate cancer cells, gnetin C was shown to have more potent MTA1-mediated cytotoxicity, apoptosis, inhibition of clonogenic cell survival, and motility compared to resveratrol and pterostilbene [31]. To evaluate gnetin C’s clinical potential, in the current study, we assessed the efficacy of gnetin C-supplemented diets as MTA1-targeted interception using a unique transgenic mouse model (*R26^MTA1^*; *Pten^+/f^*; *Pb-Cre^+^*) representing high-risk early-stage prostate cancer.

The clinical significance of MTA1 in prostate cancer progression and metastasis has been reported [28,29,30,39]. We have also demonstrated that MTA1 is a molecular target for stilbene polyphenols, such as resveratrol, pterostilbene, and gnetin C, in prostate cancer, in vitro and in vivo [22,24,25,26,34,35,40,41]. Our group has tested the MTA1-targeted chemopreventive and therapeutic potential of stilbenes and grape extracts in murine prostate cancer models [22,24,27,42]. Particularly, our recent report showed that a pterostilbene-supplemented diet fed to mice with early-stage prostate cancer can block the progression of prostate cancer through the inhibition of MTA1-mediated signaling [27]. Since gnetin C showed improved pharmacokinetic parameters in mouse and human studies compared to both resveratrol and pterostilbene [35,43,44,45,46], we sought to compare, for the first time, the MTA1-targeted inhibitory efficacy of gnetin C and pterostilbene supplemented diets in a transgenic mouse model of early-stage prostate cancer.

For this study, we fed prostate-specific *R26^MTA1^*; *Pten^+/f^* mice reference diets (Ctrl-Diet and Pter-Diet) along with gnetin C-supplemented diets, and asked two major questions: (1) whether gnetin C at the same diet concentration has a more potent MTA1-mediated effect than pterostilbene; and (2) whether gnetin C at half diet concentration has beneficial efficacy in this model.

While both pterostilbene- and gnetin C-supplemented diets showed expected beneficial effects compared to Ctrl-Diet, the differences between Pter-Diet and Gnetin C-Diet (s) were considerable: mice treated with Gnetin C-Diet(s) exhibited favorable histopathology compared to mice fed Pter-Diet. Immunohistochemical results showed a statistically significant decrease in epithelial cancer cell proliferation and angiogenesis in mice fed Gnetin C-Diet(s) compared to the Pter-Diet. Furthermore, gnetin C at both concentrations had potent anticancer activity through targeting MTA1, and this effect was statistically significant compared to the Pter_70_-Diet group. In addition, consistent with apparent trends in inhibiting MTA1 expression after gnetin C treatment, levels of PTEN were increased and p-Akt were decreased in Gnetin C-Diet groups. Further analysis of prostate tissues and prostate cancer cell lines confirmed the potent MTA1 inhibitory potential of gnetin C compared to pterostilbene. The more potent biological effects of gnetin C could be explained by its improved pharmacokinetics [44].

It has been reported that stilbenoids can regulate cytokine expression in different cellular systems [47,48,49]. With regards to cytokine-mediated anti-inflammatory effects in cancer, resveratrol was found to inhibit metastasis and angiogenesis by reducing inflammatory cytokines, such as tumor necrosis factor α (TNFα), IL-6, and interleukin 1 beta (IL-1β), in vitro and in vivo in oral cancer [50], and decreasing levels of the pro-inflammatory cytokines in rat colon carcinogenesis [51]. In prostate cancer, we have shown the inhibitory effects of orally administered resveratrol, trimethoxy-resveratrol, and piceatannol on circulating IL-6 levels in LNCaP xenografts [41]. In addition, the inhibitory effects of dietary pterostilbene on IL-6 levels were also detected in sera from *R26^MTA1^*; *Pten^+/f^* transgenic mice [27]. The inhibitory effects of grape extract diet supplementation and pterostilbene treatment on the levels of pro-inflammatory and pro-angiogenic IL-1β were also reported in prostate-specific *Pten*-deficient mouse models [26,42].

Here, murine serum was used to evaluate the systemic cytokine-mediated immune response to the gnetin C-supplemented diets. Our results show that, consistent with our previous observations [27], pterostilbene reduced the levels of IL-6 compared to the control. However, interesting results were obtained with gnetin C: low diet concentration of gnetin C (35 mg/kg diet) significantly suppressed levels of pro-inflammatory IL-2, and to a lesser extent, levels of IL-6. Curiously, the effectiveness of high-concentration gnetin C (70 mg/kg diet) was profoundly diminished for both cytokines, even resulting in a rather opposite effect by drastically increasing levels of IL-6. Our unexpected results on the systemic anti-inflammatory effects of gnetin C are consistent with some reports that natural products or individual polyphenols at lower doses exert more potent anti-inflammatory/anticancer effects than at higher doses in vivo [17,52,53,54]. Relevant to our study, a low dose of melinjo seed extract (MSE) that contains gnetin C had more profound effects on tumor growth and angiogenesis in mice with colon tumors than it did at double the dose [17]. Further studies are needed to clarify the dose-and-effect relationship of natural polyphenols as anticancer and anti-inflammatory agents.

Melinjo fruit is consumed as a food in Southeast Asia as well as being traditional medicine [55]. Importantly, nontoxic effects of MSE and gnetin C in nonmalignant cells in culture [17,36,37,56] and in vivo toxicity studies in mice [17] and rats [57] has been demonstrated. Crucially, MSE and gnetin C appear to be safe in humans, as it has been demonstrated in clinical trials [44,45,58,59].

Based on an average food consumption of 4g diet per day for each mouse (Figure 1C, middle panel), doses in the current study were 0.28 mg per day for the Gnetin C_70_ diet and Pter_70_ diet, and was 0.14 mg per day for the Gnetin C_35_ diet. There are no other in vivo studies with these compounds in transgenic mouse models, except our own studies [22,26,27]. The dose that we used in our study is in line with the safe doses used in human studies.

In summary, to the best of our knowledge, the current study is the first to demonstrate the in vivo anticancer activity of gnetin C-supplemented diets in a clinically relevant transgenic mouse model of prostate cancer. Due to the known safety of gnetin C, and its improved bioavailability compared to pterostilbene and resveratrol, lower doses of gnetin C may become one of the most promising targeted strategies for prostate cancer interception. Our study also suggests that the evaluation of pro-inflammatory cytokines in the blood may provide noninvasive prognostic and predictive biomarkers. The curious mixture of gnetin C’s ability to inhibit cancer cell proliferation and simultaneously have a complex immunomodulatory response at high concentrations needs further investigation.

## 4. Materials and Methods

### 4.1. Animals

We have previously generated prostate-specific MTA1-overexpressing mice (*R26^MTA1^*; *Pb-Cre^+^*) [27]. The generation of prostate-specific MTA1 overexpression on the background of *Pten* heterozygous mice was achieved by breeding our MTA1 transgenic mice (*R26^MTA1^*) with a C57BL/6J female mouse homozygous for the “floxed” *Pten* allele, which was purchased from Jackson Laboratories (Bar Harbor, ME, USA). Transgenic *R26^MTA1^*; *Pten^+/f^*; *Pb-Cre^+^* male mice (hereafter *R26^MTA1^*; *Pten^+/f^*) for this study were confirmed by PCR-based tail genomic DNA genotyping using primers, as previously described [27]. The animals were housed in cages with corn cob bedding in a temperature-controlled room with a 12 h light–dark cycle. Mice had free access to drinking water and designated irradiated AIN-76A diets (Envigo Teklad, Boyertown, PA, USA). All animal protocols were approved in advance by the Institutional Animal Care and Use Committee (IACUC) at Long Island University in accordance with the NIH Guidelines for the Care and Use of Laboratory Animals. Mice were monitored daily for their general health and signs of toxicity.

### 4.2. Diets and Study Design

Gnetin C was a generous gift from Hosoda SHC Co., Ltd. (Fukui, Japan). The purity of gnetin C and pterostilbene (Pter) (Sigma Aldrich, St. Louis, MO, USA) was determined to be ≥ 99%. Gnetin C and Pter powders were shipped to Envigo Teklad Diets (Boyertown, PA, USA) for the formulation of three different supplemented diets on the basis of an AIN-76A control diet (Ctrl-Diet). Based on our previous experience with a Pter diet at a concentration of 100 mg/kg diet [27], we formulated the following diets with gnetin C and pterostilbene, as a reference diet: gnetin C, high concentration, 70 mg/kg diet (Gnetin C_70_-Diet); gnetin C, low concentration, 35 mg/kg diet (Gnetin C_35_-Diet); and pterostilbene at 70 mg/kg diet (Pter_70_-Diet). For our calculations, we used the following formula: DD = (SD × BW)/FI (Research Diets Inc, New Brunswick, NJ, USA), where DD is diet dose (mg compound/kg Diet); SD is single dose (mg compound/kg bw/day); BW is body weight (g bw/animal), and FI is daily food intake (g Diet/day). For prospective clinical relevance of the doses used in this study, we determined equivalent doses for humans using the following human equivalent dose (HED) formula: HED (mg/kg) = Animal dose (mg/kg) × Km ratio, where the Km ratio for mice is 0.081 [60]. Considering that the average human male BW is 70 kg, the doses used in this study for mice approximately translate into 53.2 mg/day (Gnetin C_70_-Diet), 26.6 mg/day (Gnetin C_35_-Diet), and 53.2 mg/day (Pter_70_-Diet) in humans. All these doses are well tolerated and safe in humans, as shown in respective clinical trials [45,58,59,61]. The diets were stored at 4 °C and protected from light. Fresh diets were weighed each week and added to cages. The nutritional composition of diets was published previously [27].

*Treatment groups:* Twenty-four 3-week-old *R26^MTA1^*; *Pten^+/f^* mice were randomized into four major groups, *n* = 6 per group: Ctrl-Diet; Pter_70_-Diet; Gnetin C_70_-Diet; and Gnetin C_35_-Diet. All mice were fed ad libitum. The animals were weighed weekly and monitored regularly for their food intake and general health. After 17 weeks on their respective diet, mice were euthanized by CO_2_ inhalation and cervical dislocation. Due to small proportions of mouse prostate, animals from the same treatment group were used for different purposes. From six mice in each treatment group, we used three mice for the UGS (seminal vesicle, prostate, and the urinary bladder) isolation to be processed for histology and IHC. The remaining three mice were used for the isolation of prostate tissues for molecular analysis (protein and RNA isolation). Prostate tissues were dissected, snap frozen, and kept at −80 °C until use for further molecular analyses. Blood was collected by cardiac puncture upon sacrifice at week 17; serum samples were prepared and stored at −80 °C.

### 4.3. Histopathology and Immunohistochemistry

Urogenital system tissues were fixed in 10% neutral-buffered formalin, processed, and embedded in paraffin, cut into 4 μm sections, and mounted onto slides (Reveal Biosciences, San Diego, CA, USA). H&E-stained sections were assessed for mouse PIN and/or adenocarcinoma. Slides were subjected to IHC analysis as described previously [22,27], using antibodies from Abcam (Boston, MA, USA) for Ki67 (1:50) and SMA (1:700), and from Cell Signaling Technology (Beverly, MA, USA) for CD31 (1:500), MTA1 (1:50), PTEN (1:150), and pAkt^Ser473^ (1:50). Images were taken using an EVOS XL Core microscope (Thermo Fisher Scientific, Somerset, NJ, USA). Ki67, MTA1, and CD31 positively stained cells were quantified in five randomly selected areas using Image J software.

### 4.4. Tissue Processing and Western Blot Analysis

Frozen prostate tissues were homogenized in RIPA buffer (Thermo Fisher Scientific, Somerset, NJ, USA), and Western blots were performed as described previously [22,26,27,42]. Briefly, samples were separated using 10–15% polyacrylamide gels, and transferred onto polyvinylidene difluoride membranes. Membranes were blocked with 5% milk/TBS/0.1% Tween, and then probed with primary antibodies for MTA1 (1:2500); PTEN (1:1000); and Akt and p-Akt^S473^ (1:1000) from Cell Signaling Technology (Beverly, MA, USA). Β-actin (1:5000), Hsp70 (1:1000), and GAPDH (1:1000) were purchased from Santa Cruz Biotechnology (Dallas, TX, USA), and were used as loading controls. Signals were detected using enhanced chemiluminescence (Thermo Fisher Scientific, Somerset, NJ, USA). Band intensity was measured using Image J.

### 4.5. Tissue Processing and Real-Time RT-PCR

Total RNA was isolated from prostate tissues that were stored in RNAlater (Thermo Fisher Scientific, Somerset, NJ, USA) immediately after tissue extraction using an miRNeasy mini kit (Qiagen, Germantown, MD, USA) as recently described [27]. The quality of RNA was evaluated on a NanoDrop spectrophotometer (Shimadzu Scientific Instruments, Kyoto, Japan). Quantitative RT-PCR was performed on a Lightcycler 480 II Real-Time PCR instrument (Roche Diagnostics, Indianapolis, IN, USA) using murine primers specific for MTA1 (forward: 5′-AGC TAC GAG CAG CAC AAC GGG GT-3′; reverse: 5′-CAC GCT TGG TTT CCG AGG AT-3′) and PTEN (forward: 5′-GAT TAC AGA CCC GTG GCA CT-3′; reverse: 5′-GGG TCC TGA ATT GGA AT-3′. β-actin was used for normalization (forward: 5′-CGT GGG CCG CCC TAG GCA CCA-3′; reverse: 5′-TTG GCT TAG GGT TCA GGG GGG-3′) (Integrated DNA Technologies, Coralville, IA, USA). Fold changes in mRNA expression were estimated by the 2^−ΔΔCt^ method.

### 4.6. ELISA

Serum IL-2 levels were analyzed using commercially available mouse IL-2 and IL-6 ELISA kits (Abcam, Boston, MA, USA) as per the manufacturer’s instructions. Samples (50 μL) or standards were added to the pre-coated 96-well strip microplates covered with an anti-tag antibody, followed by the antibody mix, and incubated for 1 h at room temperature. After washing, 3, 3′,5,5′-tetramethylbenzidine substrate was added for 10 min at room temperature, followed by the stop solution, to each well. The reaction was read at 450 nm using a Tecan Sunrise Absorbance microplate reader (Tecan, Mannedorf, Switzerland). Using the standard titration curve, a concentration of IL-2 and IL-6 in serum was calculated based on their absorbance.

### 4.7. Cell Culture, Reagents, and Treatment

Prostate cancer 22Rv1 cells (ATCC, Manassas, VA, USA) were grown and maintained in RPMI-1640 media containing 10% FBS, as described previously [27,62]. Cells were validated for mycoplasma-free condition using the Universal Mycoplasma Detection Kit (ATCC, Manassas, VA, USA). Gnetin C was a generous gift from Hosoda SHC Co., Ltd. (Fukui, Japan). Pterostilbene was purchased from Sigma-Aldrich (St. Louis, MO, USA). Compounds were dissolved in pure dimethyl sulfoxide (DMSO, 0.1% final concentration) and stored in the dark at −20 °C until use. At approximately 60% confluency, cells were treated with pterostilbene and gnetin C at the same concentration of 25 µM for 24 h, after which, protein lysates were isolated for Western blot analysis, as described above. The dilutions for antibodies used in cell lines were the same as those used for tissues.

### 4.8. Statistical Analysis

The data from each group of mice were summarized as the mean ± SD/SEM. The statistical significance of differences between groups was determined by a one-way ANOVA (Prism v9, GraphPad Software, San Diego, CA, USA). A *p*-value of ≤ 0.05 was considered statistically significant.

## 5. Conclusions

In conclusion, our results established the MTA1/PTEN/p-Akt axis as a suitable target for gnetin C interception in early-stage prostate cancer in mice. The clinical usage of gnetin C may be a valuable tool for the management of prostate cancer progression in selected patients under active surveillance. We intend to use synthetic chemistry to produce gnetin C analogs with higher potency that can be used as pharmaceuticals. The development of natural and synthetic MTA1 inhibitors with improved pharmacokinetic profiles will secure the utilization of these new and targeted drugs not only for interception during early-stage prostate cancer, but also in the treatment of more advanced stages and metastatic disease.

## Data Availability

Not applicable.

## Supplementary Materials

The following supporting information can be downloaded at: https://www.mdpi.com/article/10.3390/cancers14246038/s1, Figure S1: Screening of prostate tissues for molecular markers from mice fed different diets; Figure S2: Effects of diets supplemented with Gnetin C on the levels of circulating inflammatory IL-6 cytokine detected in murine serum by ELISA; Figure S3: Gnetin C inhibits proliferation of prostate cancer cells in a dose-dependent manner.

## Abbreviations

**Akt**	v-Akt murine thymoma viral oncogene (protein kinase B)
**ANOVA**	Analysis of variance
**AR**	Androgen receptor
**CD31**	Cluster of differentiation 31
**DMSO**	Dimethyl sulfoxide
**DNA**	Deoxyribonucleic acid
**DU145**	Human prostate cancer cell line
**Ctrl-Diet**	Control Diet
**ELISA**	Enzyme-linked immunosorbent Assay
**ETS2**	ETS proto-oncogene 2, transcription factor
**GAPDH**	Glyceraldehyde 3-phosphate dehydrogenase
***^+/f^***	*Pten* gene flanked by two loxP sites in one allele
**Gnetin C_70_-Diet**	Gnetin C high dose diet (70 mg/kg)
**Gnetin C_35_-Diet**	Gnetin C low dose diet (35 mg/kg)
**H&E**	Hematoxylin and eosin
**Hsp70**	Heat shock protein 70
**IACUC**	Institutional Animal Care and Use Committee
**IHC**	Immunohistochemistry
**IL-1β**	Interleukin 1 beta
**IL-2**	Interleukin 2
**IL-6**	Interleukin 6
**kCal**	Kilocalorie
**Ki67**	Cellular protein marker of proliferation
**LIU**	Long Island University
**LNCaP**	Human prostate cancer cell line
**MSE**	Melinjo seed extract
**mRNA**	Messenger RNA
**MTA1**	Metastasis-associated protein 1
**Notch2**	Transmembrane protein
**NS**	Nonsignificant
**NuRD**	Nucleosome remodeling and deacetylase complex
**p-Akt**	Phosphorylated Akt
**p-Akt^S473^**	Phosphorylation of serine 473 in C-terminus of Akt
**Pb-Cre^+^**	Probasin promoter directing expression of epithelial Cre recombinase
**PC3**	Human prostate cancer cell line
**PC3M**	Human prostate cancer cell line
**PCR**	Polymerase chain reaction
**PIN**	Prostate intraepithelial neoplasia
**PTEN**	Phosphatase and tensin homolog
***Pten* ^+/f^**	*Pten* heterozygous mice
**Pter**	Pterostilbene
**Pter_70_-Diet**	Pterostilbene 70 mg/kg diet
**PVDF**	Polyvinylidene fluoride
**qRT-PCR**	Quantitative reverse transcriptase polymerase chain reaction
**RNA**	Ribonucleic acid
**RPMI-1640**	Roswell Park Memorial Institute 1640 cell culture media
**RWPE-1**	Immortalized human prostatic epithelial cell line
**22Rv1**	Human prostate cancer cell line
**R26**	Rosa26 loci in the mouse genome
**SD**	Standard deviation
**SEM**	Standard error of mean
**SMA**	Smooth muscle actin
**TNFα**	Tumor necrosis factor alpha
**UGS**	Urogenital system
