# Supplementary material for: Gnetin C Intercepts MTA1-Associated Neoplastic Progression in Prostate Cancer
Source: Cancers (Basel). 2022 Dec 8;14(24):6038. doi: 10.3390/cancers14246038 (PMC9775406; doi:10.3390/cancers14246038)
Supplement: Supplementary file 1 [file cancers-14-06038-s001.zip › cancers-1998561-supplementary.pdf]

Supplementary Information:

"MTA1-mediated efficacy of Gnetin C-supplemented diet in transgenic mouse model of prostate cancer"

Prashanth Parupathi, Gisella Campanelli, Rabab Al Deabel, Anand Puaar, Lakshmi Sirisha Devarakonda, Avinash Kumar, and Anait S Levenson

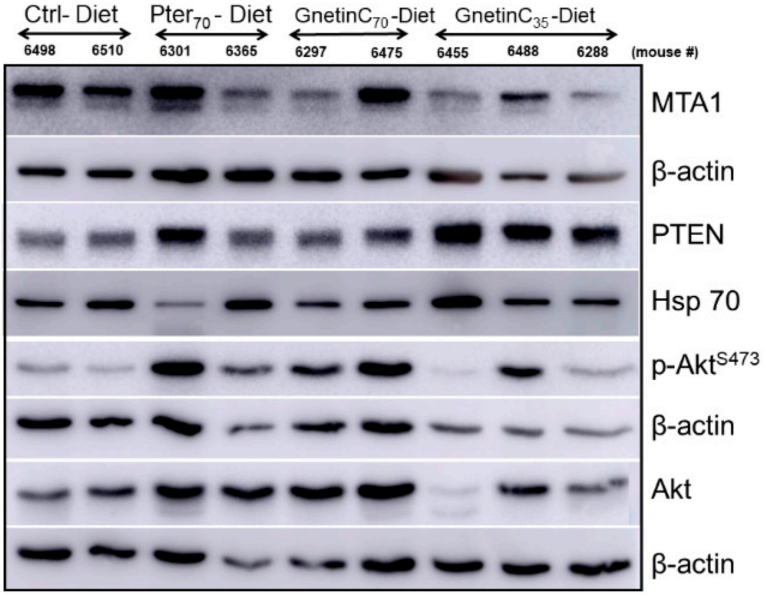

Figure S1. Screening of prostate tissues for molecular markers from mice fed different diets. Gnetin C_{35}-Diet treated mice consistently showed inhibition of MTA1 and associated p-Akt/Akt and upregulation of PTEN. Other treatment groups demonstrated more heterogeneity in response to compounds, when compared to Ctrl mice. Western blot was performed as described in *Material and Methods*.

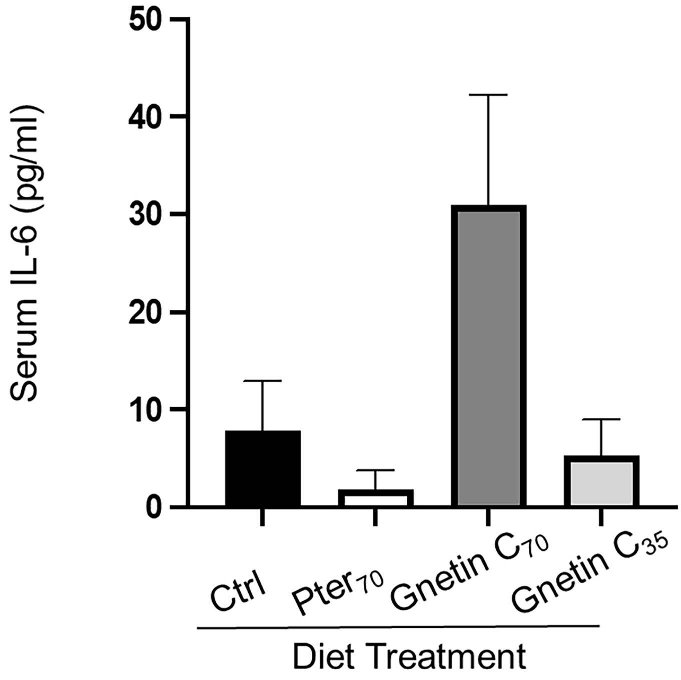

Figure S2. Effects of diets supplemented with Gnetin C on the levels of circulating inflammatory IL-6 cytokine detected in murine serum by ELISA. Circulating levels of IL-6 in sera collected from precancerous *R26*^{MTA1}; *Pten*$^{f/+}$; *Cre*$^{+}$ mice on different diets (n = 2 per group). Data represents the mean ± SEM of two independent experiments performed in duplicates. p = 0.075, ns between groups (one-way ANOVA).

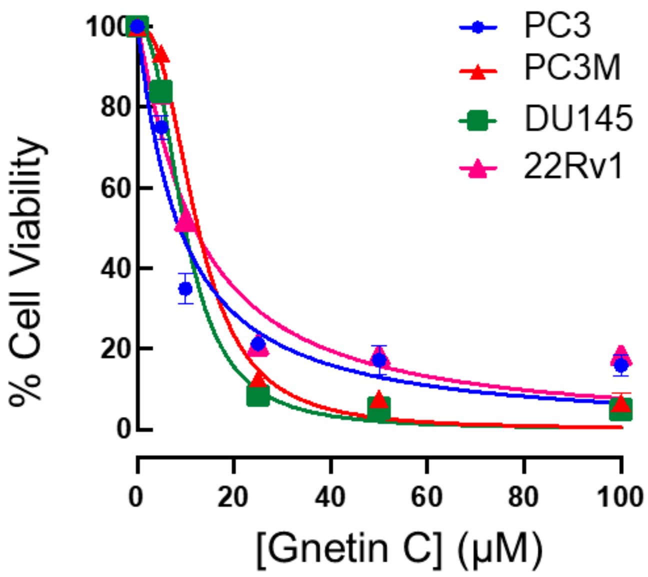

Cell line	IC_{50}
PC3	8.78
PC3M	12.59
DU145	9.88
22RV1	11. 86

Figure S3. Gnetin C inhibits proliferation of prostate cancer cells in a dose-dependent manner. MTT cell viability assay was performed using PC3, PC3M, DU145, and 221Rv1 prostate cancer cell lines as described earlier [25, 29, 30, 34]. IC_{50} was calculated using GraphPad Prism v9 software.
